# Assessing the effectiveness of the transformational coaching workshop using behavior change theory

**DOI:** 10.1177/17479541221122435

**Published:** 2022-08-30

**Authors:** Caroline Hummell, Jordan D Herbison, Jennifer Turnnidge, Jean Côté

**Affiliations:** Reviewers: Andy Gillham (Sanford Sports Science Institute, USA) Thelma Horn (Miami University, OH, USA); 1 104266 Brock University, St. Catharines, Ontario, Canada; 2 5620 McGill University, Montreal, Quebec, Canada; 3 152964 Queen’s University, Kingston, Ontario, Canada

**Keywords:** Leadership, motivation, psychosocial development, youth sport

## Abstract

The current study assessed how participation in the Transformational Coaching Workshop (TCW) influenced youth sport coaches’ perceived capability, opportunity, and motivation to incorporate transformational coaching behaviors into their coaching practices. Sixty-three volunteer youth sport coaches participated in the study as part of an intervention (*n* = 31; *M_age_* = 45.65 years; *SD_age_* = 8.82 years) or comparison group (*n* = 32; *M_age_* = 44.59 years; *SD_age_* = 11.86 years). The study employed a two-arm, pre- and post-intervention, non-randomized intervention design. Dependent- and independent-sample *t*-tests were conducted to assess within and between-group differences. Results indicated that participants in the intervention group reported slight improvements in their perceived capability and opportunity to use transformational coaching behaviors post-intervention. There were no significant differences between groups post-intervention. This study provides support for the effectiveness of the TCW, and the application of behavior change frameworks to evaluate coach development programs.

Quality coach–athlete relationships are a key determinant of youths’ positive sport experiences.^1^ Previous research shows that coach–athlete relationships can foster athletes’ intrinsic motivation,^2^ passion for sport,^3^ and satisfaction with training and performance.^4^ Despite this research, the mechanisms by which coach–athlete relationships are positively influenced by coaches’ interpersonal knowledge and behaviors remain limited.^5^ One way to help coaches better understand how their behaviors affect their interactions with athletes is through Coach Development Programs (CDPs). CDPs are educational interventions that use activities (e.g., video clips and personal reflection) to help coaches improve their attitudes and knowledge of the behaviors that are known to positively influence coach–athlete relationships.^6^ Consequently, it is important to evaluate the impact that CDPs have on how coaches perceive interpersonal knowledge and behaviors in real-world sport settings.

Through a review of existing CDPs, Allan et al.^7^ noted that interpersonal-focused CDPs, such as Coach Effectiveness Training,^8,9^ Mastery Approach to Coaching (MAC),^10^ and Empowering Coaching™,^11^ primarily utilize motivational frameworks (e.g., Achievement Goal Theory^12^; Self-Determination Theory (SDT^13^)) to enhance coaches’ understanding of the interpersonal behaviors that are associated with positive athlete outcomes. However, CDPs informed by motivational theories include content that focuses on athletes’ outcomes and the *what* of coaches’ behaviors (i.e., instruction and feedback) but provide limited content that directly and systematically addresses the interpersonal nature of coaching or the *how* of coaches’ behaviors (e.g., the interpersonal element of delivering content). Given that previous research supports how specific interpersonal coaching behaviors (e.g., discussing personal matters) impact the psychosocial development of athletes,^14,15^ researchers have looked to frameworks that explain *how* coaches can use interpersonal behaviors effectively.

Transformational Leadership (TFL) is a person-centered leadership approach that aims to positively foster follower development.^16^ The conceptualization of TFL proposed by Bass and Riggio^16^ involves four dimensions referred to as the 4 Is: (a) Idealized Influence, (b) Inspirational Motivation, (c) Intellectual Stimulation, and (d) Individualized Consideration. Studies indicate that sport coaches who use TFL behaviors improve sports performance via the mediating effects of intrinsic motivation.^17^ Furthermore, coaches’ use of TFL is linked with developmental outcomes in youth athletes, such as the growth of personal, social, and cognitive skills^18^ and influencing task and social cohesion in team sports.^19^

Turnnidge and Côté^20^ recently identified 11 TFL behaviors situated within the 4 Is of TFL (see Table 1). These behaviors were developed through an iterative process involving literature reviews of behavioral observation instruments, interviews with youth sport coaches, reviews of existing questionnaires, behavioral observation systems, and TFL and coaching literature.^20^ These 11 behaviors have become the foundation of a CDP known as the Transformational Coaching Workshop (TCW).^21^ Through video observation and coding using the Coach Leadership Assessment System,^20^ Lawrason et al.^22^ found that the observable leadership behaviors of coaches changed following participation in the TCW; however, the authors noted an uncertainty of what factors contributed to the changes post-workshop. As such, it is unclear whether the intervention affects participants’ perceptions related to implementing TFL behaviors in their daily coaching practices. Whitley et al.^23^ put out a call for researchers to begin testing conditions and mechanisms identified in intervention theories as opposed to the outcomes of interventions. Accordingly, sports researchers suggest that using behavior change theory, such as the COM-B model (i.e., Capability, Opportunity, Motivation-Behavior (COM-B) model^24^), is a suitable solution to more comprehensively examine how CDPs promote observable changes in coaches’ behaviors.^7,22^

**Table 1.** Summary of the transformational coaching behaviors.

Higher order dimension (“I”)	Leadership behavior
Idealized influence	1. Discussing and modeling pro-social values or behaviors
	2. Showing vulnerability and humility
Inspirational motivation	3. Discussing goals and expectations
	4. Expressing confidence in athlete(s)’ capabilities
	5. Implementing a collective vision
	6. Providing meaningful and challenging tasks and roles
Intellectual stimulation	7. Eliciting athlete input
	8. Sharing decision making and leadership responsibilities
	9. Emphasizing the learning process
Individualized consideration	10. Showing interest in athletes’ needs
	11. Recognizing individual roles and contributions

The COM-B model suggests that there are three elements required to change a person's behavior*:* (a) capability (i.e., an individual's physical or psychological ability to execute the preferred behavior); (b) opportunity (i.e., the physical or social environment that can enable an individual's behavior), and (c) motivation (i.e., an individual's internal automatic or reflective processes that direct these behaviors^24^). The COM-B model has previously been applied in health promotion research to explore barriers and enablers to the delivery of a health assessment program for Australian preschool children,^25^ the self-care behaviors of individuals with chronic heart failure,^26^ and the usability of smartphone-connected listening devices in adults with hearing loss,^27^ to name a few. However, the COM-B model has yet to be applied to examine the effectiveness of a CDP for youth sport coaches specifically. According to Michie et al.,^24^ how individuals perceive their capability, opportunity, and motivation to perform a behavior directly influences behavior change. Furthermore, these three factors interact so that behavior change is part of a dynamic system with positive and negative feedback loops. Therefore, it is important to understand CDP features that impact coaches’ perceptions of their capability, opportunity, and motivation to use transformational coaching behaviors after participation in the TCW.

In sum, previous research shows that the TCW has facilitated a change in youth coaches’ observable leadership behaviors.^22^ However, it remains unclear what effect the TCW has on coaches’ perceptions of the three elements identified as necessary for behavior change. Thus, the purpose of this study was to assess the TCW's effect on youth sport coaches’ perceived capability, opportunity, and motivation to use transformational coaching behaviors to provide an understanding of why coaches show observable changes in behavior post-workshop (i.e., COM-B model^24^). It was hypothesized that coaches who participated in the intervention would show an increase in their post-intervention scores for perceived capability, opportunity, and motivation to use TFL behaviors when (a) compared to their pre-intervention scores, and (b) compared to coaches who did not participate in the intervention at post-intervention.

## Method

### Design

The study used a quasi-experimental pre-post design for unequal groups as this research took place in a naturalistic setting (i.e., coaching context) where the random assignment of participants or manipulation of the independent variable was not possible.^28^ Furthermore, unforeseen circumstances, such as the onset of the COVID-19 pandemic, also impacted the researcher's ability to control for the random assignment of participants in the present study. Nonetheless, a quasi-experimental approach allows for the strengthening of causal inferences while maintaining internal and external validity without interrupting the “real life” of the participants through intrusive intervention.^28,29^ Therefore, participants for this study were part of either an intervention group (i.e., coaches who participated in the TCW) or a comparison group (i.e., coaches who did not participate in the TCW).

### Participants

A priori power analysis using G*Power3^30^ calculated the sample size needed to test both the difference between two dependent group means and two independent group means with a medium to large effect size (Cohen's *d* = 0.60) and a 95% confidence interval (alpha of 0.05). Results showed that a total sample of 54 participants would be needed (27 per group) to explore significant within-group differences, and 102 participants would be required (51 per group) to explore significant between-group differences to achieve a power of 0.80.

Sixty-three male (*n* = 51) and female (*n* = 12) volunteer youth sport coaches from Ontario, Canada, took part in the study as part of the intervention (*N* = 31; *n_male_* *=* *30; n_female_* *=* *1; M_age_* = 45.65 years; *SD_age_* = 8.82; *M*_experience_ = 16.06 years; *SD_experience_* = 11.40) or comparison group (N = 32; *n_male_* *=* *21; n_female_* *=* *11; M_age_* = 44.59 years; *SD_age_* = 11.86; *M*_experience_ = 12.53 years; *SD_experience_* = 10.79). No significant differences were found across participants for age, gender, or years of experience coaching for any of the study variables tested. The inclusion criteria for this study required coaches to coach youth (i.e., ages 12–18 years), be a volunteer (i.e., not paid), have a minimum of one year of experience coaching, and be currently coaching at the time of participation in the study. In addition, participants had earned a variety of coaching certifications including National Coaching Certification Program levels 1–3 (*n* = 45), Respect in Sport (*n* = 15), and Gender Identity and Expression (*n* = 4). These programs indirectly refer to interpersonal coaching behaviors; however, they seldom explain how or when to use specific interpersonal coaching behaviors in practice. Consequently, none of the coaches reported receiving specialized training to enhance their interpersonal behaviors specifically.

### Measures

Participants completed a sport-adapted COM-B questionnaire pre- and post-intervention. The sport-adapted COM-B questionnaire was developed by Rochon et al.^31^ based upon recommendations by Michie et al.^32^ for creating COM-B measures and Huijg et al.^33^ theoretical domains framework questionnaire for evidence-informed research. It includes 52 items divided into four subsections that correspond to each component in the COM-B model. Specifically, the subsections measure 13 items for coaches’ perceptions of their physical capability (e.g., “I have the necessary knowledge and understanding to elicit athlete input”), 13 items of psychological capability (e.g., “I have the interpersonal skills to elicit athlete input”), 13 items for opportunity (e.g., “I have the necessary resources to elicit athlete input”), and 13 items for motivation (e.g., “I want to elicit athlete input”) to perform the 11 transformational coaching behaviors (for a review of these behaviors, see Ref. 20). Responses fall on a 7-point Likert-type scale ranging from 1 (*strongly disagree*) to 7 (*strongly agree*); thus, higher scores reflect higher perceptions of physical capability, psychological capability, opportunity, and motivation to use transformational coaching behaviors. Previous research using the same sport-adapted COM-B questionnaire^34^ to evaluate the TCW in the parasport context demonstrated excellent internal consistencies for the COM-B questionnaire, with values of α = 0.97 for the 26 items related to capability, α = 0.94 for the 13 items related to opportunity, and α = 0.89 for the 13 items related to motivation. For the present study, internal reliability was excellent at both time points for capability (*a_T1_* = .96; *a_T2_* = .95), opportunity (*a_T1_* = .95; *a_T2_* = .92), and motivation (*a_T1_* = .90; *a_T2_* = .93). The questionnaire maintained content validity as items were informed by the recommendations of Michie et al.^32^ and tested in two previous studies (i.e., Refs. 31,34). Face validity was achieved as the items included in the questionnaire were developed, reviewed, and approved by experts familiar with the design of behavior change research. Such experts included a Tier 2 Canada Research Chair in Physical Activity Promotion and Disability and a Health Education Research Associate at a top Canadian research-intensive institution.

### Procedure

**Figure 1.**
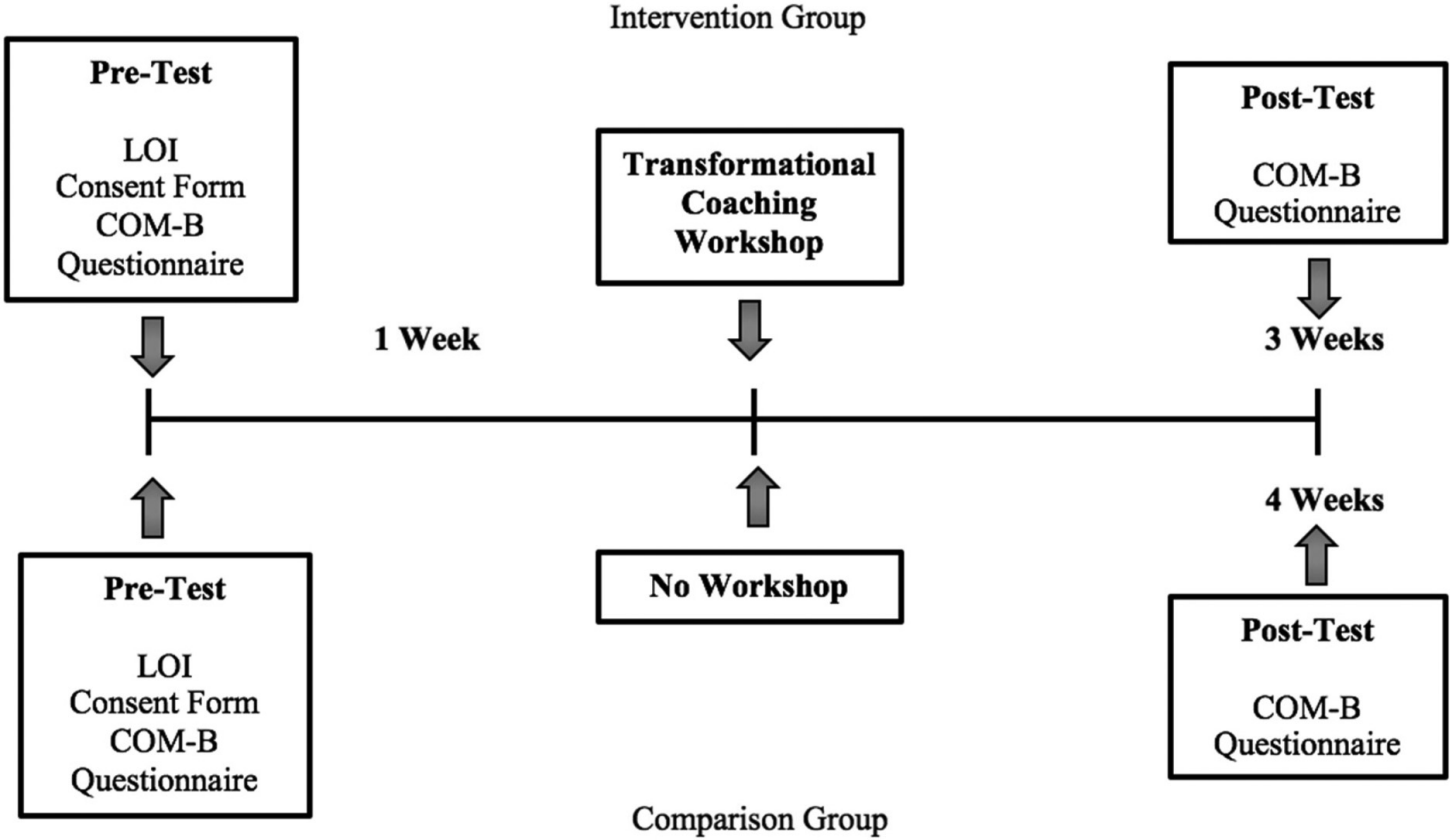
Procedural timeline for data collection. *Note*. LOI = letter of intent, COM-B = capability-opportunity-motivation.

Following ethics approval, recruitment e-mails were sent to local youth sports organizations to inform coaches about the study. Participant recruitment occurred in two waves. From July 2019 to March 2020, youth sport coaches were recruited to participate in the intervention group. The TCW was offered based on the organizations’ schedules and coaches’ availability. Due to unanticipated lockdown measures because of the COVID-19 pandemic, recruitment for the intervention group ceased in early March 2020 as sports programing was no longer running and coaches would be unable to practice learned transformational coaching behaviors post-workshop. Recruitment for the comparison group occurred between late February 2020 and April 2020. Coaches who initially expressed interest in attending the TCW but were no longer able to participate due to the unforeseen lockdown measures resulting from the onset of the COVID-19 pandemic were assigned to the comparison group. Additionally, the comparison group included coaches from youth sports organizations in Ontario who closely matched the demographics of the intervention group participants (e.g., type of sport and age) and who were unable to attend the workshop pre-pandemic due to other circumstances (e.g., geographical location and scheduling conflicts). While in-person sports programing stopped during the data collection period for the comparison group, this did not affect the coaches’ abilities to report on their perceptions of the transformational coaching behaviors as they did not attend the TCW and therefore did not need an opportunity to practice the learned behaviors. Once the number of coaches within the comparison group matched the number of coaches within the intervention group, recruitment for the study ceased. For a complete visualization of the procedural timeline, please see Figure 1.

#### Pre-intervention procedure

One week before participating in the TCW, participants in the intervention group completed the pre-intervention COM-B questionnaire package, including the letter of information and consent form. Participants were given the option to complete online or paper versions of the questionnaire package. A total of seven coaches completed questionnaire packages on paper, while the remaining 24 coaches completed the questionnaires electronically through a Qualtrics™ link distributed via e-mail. Consent forms and questionnaires were collected between seven days before and the day of the TCW. The questionnaires took approximately 20 minutes to complete. Participants in the comparison group were sent the pre-intervention COM-B questionnaire package within 24 hours of agreeing to participate in the study. The completion date of pre-intervention measures was kept on file to determine when the post-intervention questionnaire package would be administered.

#### Workshop

Coaches in the intervention group participated in the TCW seven days after completing the pre-intervention questionnaires. The TCW, a one-time intervention, takes approximately 4 hours to complete and follows the same procedures described in previous studies (for a complete description of the TCW, please see Ref. 21). Before beginning the TCW, coaches received a workbook containing activities and information to be reviewed during the workshop and a notebook for participants’ additional notes. Using a variety of activities, discussions, and videos, coaches were able to observe and reflect on the principles of TFL. Overall, the TCW content and supplementary resources strategically target the way coaches perceive their use of transformational coaching behaviors.

#### Post-intervention procedure

Intervention participants received the post-intervention questionnaire package exactly three weeks post-intervention. This three-week window provided participants with an opportunity to incorporate learned TFL behaviors into their coaching practice. Participants in the comparison group were administered the post-intervention questionnaire package four weeks after completing the pre-intervention questionnaire package to mirror the data collection timeline of the intervention group. Apart from the seven participants from the intervention group who completed the pre-intervention questionnaire package on paper, all remaining participants from the intervention group (*n* = 24) and the comparison group (*n* = 32) completed the post-intervention questionnaire packages via a Qualtrics™ link. Questionnaire completion took approximately 20 minutes.

### Data analysis

#### Data cleaning

Data at both time points were determined to be missing at random (Little's MCAR test *p* > 0.90^35^). After replacing the missing values using the series mean, participants’ scores were aggregated to produce average pre- and post-intervention scores for each dependent variable (i.e., physical capability, psychological capability, opportunity, and motivation^36^). In addition, physical capability and psychological capability scores were combined to produce one aggregated score for capability, resulting in three dependent variables for the final analysis (i.e., capability, opportunity, and motivation). Pre- and post-intervention average scores for each dependent variable were then subjected to tests of normality, outliers, and descriptive statistics.

#### Pre- to post-test analyses

A Multivariate Analysis of Variance was originally opted for to address the research objectives; however, due to the data violating three out of the four assumptions tests (i.e., Shapiro-Wilk, homogeneity of regression slopes, and linearity of the covariate effect and absence of covariate by group interaction), *t*-tests were utilized to assess the within and between-group differences. More specifically, dependent-sample *t*-tests were conducted to compare the differences between the pre- and post-intervention scores for each COM-B variable in each group. Independent-samples *t*-tests were then used to compare pre- and post-intervention differences between the intervention and comparison group on each COM-B variable to observe if differences between groups occurred at either timepoint. Thus, a total of six dependent-samples *t*-tests and six independent-samples *t*-tests were conducted on the COM-B questionnaire data. Given the smaller sample, *p-*values and effect sizes were used to determine the impact of the TCW intervention and to conduct null-hypothesis significance testing. Effect sizes were calculated using Cohen's *d* with 0.2 considered small, 0.5 considered medium, and 0.8 considered large,^37^ with a significance cut-off of *p* = 0.01 after a Bonferroni adjustment was applied to account for multiple comparisons performed on the same data set.^38,39^

## Results

Table 2 displays the means and standard deviations for each of the three COM-B variables (pre- and post-intervention) for coaches in the intervention group (*n* = 31). Both the pre-intervention motivation and post-intervention motivation variables had a high negative skew, indicating that mean and median scores were less than the mode of the data set (i.e., −4.50 and −3.60, respectively); thus, a reflective transformation was applied to reduce the negative skewness of both pre- and post-intervention motivation variables before running the main statistical analyses. After achieving normality, mean scores for each capability, opportunity, and motivation variable increased after coaches participated in the TCW. While results from the dependent-sample *t*-tests appeared to show significant increases in scores from pre- to post-intervention for capability, *t* (30) = −2.07, *p* = 0.02, *d* = 0.53 (one-tailed) and opportunity, *t* (30) = −1.96, *p* = 0.03, *d* = 0.51 (one-tailed), a Bonferroni adjustment (i.e., *p* = 0.01) unveiled that the increases were not statistically significant. However, both perceived capability and opportunity exhibited medium to large effect sizes, supporting the TCW's positive influence on these variables. There were also no significant differences found from pre- to post-intervention for motivation *t* (30) = −0.41, *p* = 0.34, *d* = 0.11 (one-tailed), with a small effect size.

**Table 2.** Results of the dependent-sample t-tests for the intervention and comparison groups.

	Pre-intervention	Post-intervention			
COM-B factor	*M(SD)*	*M(SD)*	*t*	*p*	*d*
*Intervention group*					
Capability	5.86 (0.64)	6.15 (0.46)	−2.07	0.02	0.53
Opportunity	5.60 (0.85)	5.98 (0.61)	−1.96	0.03	0.51
Motivation	6.61 (0.45)	6.66 (0.41)	−0.41	0.34	0.11
*Comparison group*					
Capability	6.19 (0.65)	6.24 (0.59)	−0.48	0.32	0.01
Opportunity	5.91 (0.76)	6.01 (0.75)	−0.78	0.22	0.14
Motivation	6.63 (0.49)	6.64 (0.45)	−0.21	0.42	0.03

*Note*. *M* = mean. *SD* = standard deviation. *t* = t-value. *p* = *p*-value. *d* = effect size. COM-B factor scores range from 1 (strongly disagree) to 7 (strongly agree).

Table 2 also displays the means and standard deviations for each of the three COM-B variables (pre- and post-intervention) for coaches in the comparison group (*n* = 32). Mean scores for each capability, opportunity, and motivation variable also displayed slight increases from pre- to post-intervention. However, the results from the dependent-samples *t*-tests revealed no significant differences between scores on the COM-B variables from pre- to post-intervention within the comparison group for capability *t* (30) = −0.48, *p* = 0.32, *d* = 0.01 (one-tailed), opportunity *t* (30) = −0.78, *p* = 0.22, *d* = 0.14 (one-tailed), or motivation *t* (30) = −0.21, *p* = 0.42, *d* = 0.03 (one-tailed). Each variable also reported small effect sizes. These findings align with the first hypothesis as pre- to post-test differences in the comparison group were not expected given that no intervention was provided.

Prior to running the post-intervention comparisons, independent-sample *t-*tests were first conducted on the baseline data between the intervention group (*n* = 31) and the comparison group (*n* = 32). After the Bonferroni adjustment (i.e., *p* = 0.01), independent-samples *t*-tests revealed that there were no significant differences between the groups on baseline scores for capability *t* (61) = 2.05, *p* = 0.02, *d* = 0.51 (one-tailed), opportunity *t* (61) = 1.56, *p* = 0.06, *d* = 0.39 (one-tailed), and motivation *t* (61) = 0.096, *p* = 0.46, *d* = 0.02 (one-tailed). Similarly, no significant differences were found between the intervention and comparison group at post-intervention for capability *t* (61) = 0.701, *p* = 0.24, *d* = 0.17 (one-tailed), opportunity *t* (61) = 0.220, *p* = 0.41, *d* = 0.06 (one-tailed), and motivation *t* (61) = 0.104, *p* = 0.46, *d* = 0.03 (one-tailed).

## Discussion

The purpose of this study was to explore the TCW's^21^ influence on coaches’ perceptions of factors that influence behavior change. More specifically, this study assessed changes in youth sport coaches’ perceived capability, opportunity, and motivation to use transformational coaching behaviors following their participation in the TCW. Although perceived capability and opportunity among the intervention group participants were not significant from pre- to post-intervention following a Bonferroni correction, medium to large effect sizes were present for both variables (i.e., *d_capability_* = .53 and *d_opportunity_* = 0.51, respectively). These effect sizes suggest that the TCW had a meaningful impact on coaches’ perceived capability and opportunity to use transformational coaching behaviors. Overall, this study is the first to provide preliminary evidence of changes in coaches’ perceived capability and opportunity to use transformational coaching behaviors following the TCW, and it provides some support for the effectiveness of existing TFL-informed coaching programs.^18,22^

Existing research supports the efficacy of coaching education programs for influencing coaches’ perceived capability to use behaviors that promote positive athlete outcomes.^40,41,42^ For instance, Falcão et al.^41^ implemented a training program for coaches, modeled after the MAC intervention,^10^ and found that the program enhanced coaches’ abilities to promote life skills through sport, better understand their players, and improve their relationships with other coaches. Furthermore, Malete and Feltz^42^ found that after participating in the Program for Athletic Coach Education intervention, coaches perceived an increase in their capability to influence athletes’ learning and performance compared to pre-intervention measures. The current study aligns with previous coaching interventions and the behavior change literature, emphasizing that training can enhance the knowledge and skills that impact an individual's perceived capability to perform a behavior.^24^

Coaches believed that they had more opportunities to demonstrate transformational coaching behaviors after participation in the TCW, which is important because coaches may be more willing to apply the learned transformational coaching behaviors if they believe they have more chances to do so. Based on the COM-B model, an increase in the perception of opportunities after the workshop may link to activities in the TCW that reduced the influence of factors such as pressures from individuals (e.g., peers and parents) and organizations.^24^ For instance, Rochon^31^ found that coaches’ perceived opportunity for transformational coaching behaviors was higher if club values supported the principles of transformational coaching. Each workshop in the current study was facilitated for a particular organization, and participation was strongly encouraged and valued by the heads of the organization. Therefore, this reflects the support of club values and social support as coaches were in an environment where they were learning and practicing transformational behaviors among peers from the same organization.

Despite the large effect sizes for participants’ perceived capability and opportunity following the TCW, the results did not show a significant change in participants’ perceived motivation to use transformational coaching behaviors. The slight change in perception of motivation may be attributable to participants’ high perceived motivation in both pre- and post-intervention, which was apparent in both the intervention and comparison groups and represents a potential ceiling effect. These findings are consistent in the coaching research as other studies have shown that youth sport coaches are generally very motivated to use coaching behaviors that promote positive youth development.^43,44^ Further, the lack of significant change in participants’ perceived motivation may be due to the challenges of objectively measuring self-motivation. Past research has examined coaches’ and athletes’ perceptions of motivational climates in sport using instruments such as the Multidimensional Motivational Climate Observation System (MMCOS),^44^ but limited research exists that explores coaches’ perceptions of their self-motivation to use leadership behaviors. To date, two scales have been developed to measure coach-specific motivation.^45,46^ However, these scales measure motivation through the principles of SDT, and they have not been used to explore changes in coaches’ motivation from pre- to post-intervention. Furthermore, one study using the COM-B model found it difficult to measure certain reflective motivation constructs, such as identity, beliefs, intentions, and goals, because participants found them too abstract.^47^ Thus, researchers suggest that these deep-rooted thoughts can potentially limit the impact of an intervention and its corresponding evaluations. Moving forward, comprehensive revisions to existing measurement instruments, or the development of newer, evidence-informed instruments, may help mitigate potential limitations concerning an inability to explicitly capture changes in perceived motivation to display a certain behavior.

No significant differences were found between the intervention and comparison groups post-intervention for either capability, opportunity, or motivation variables. A lack of significant findings is likely attributable to the sample size being too small to detect such differences. Although, findings of within-group differences without between-group differences have been observed in other coaching intervention studies.^18,48^ For instance, using a similar quasi-experimental design, Vella et al.^18^ conducted an evaluation of a transformational leadership training program whereby coaches participated either as an intervention group who took part in the program or a naturalistic comparison group who received no training (i.e., comparison group). The evaluation found that athletes of coaches in the intervention group reported significant increases in their cognitive skills scores on the Youth Experience Survey for Sports^49^ from baseline to follow-up. Yet, no significant differences were found between the cognitive skills scores reported by athletes of coaches in the active group and the comparison group at the post-test. Furthermore, in the evaluation of the Empowering Coaching workshop,^48^ Solstad et al. reported that no significant differences were present between coaches’ perceived use of empowering and disempowering behaviors when the intervention group was compared to the control group in the post-test. Findings of coaches’ interpersonal interventions call for more empirical data on the effectiveness of training sessions, particularly when understanding the underlying factors of positive behavioral change in youth sport coaches.

### Limitations and future directions

Although this study has certain strengths, some limitations will be considered. First, the latter part of this research took place during the onset of the COVID-19 pandemic. The unanticipated lockdown measures and cessation of all in-person events consequently prevented the research team from hosting additional workshops (e.g., two workshops with a projected 25 coaches per workshop were canceled). As such, the small number of participants involved limited the heterogeneity and generalizability of the results and prevented the application of more robust statistical analyses (e.g., multivariate tests). While multivariate tests were the preferred analysis for this study, the data were not able to pass three out of the four assumptions and *t*-tests had to be performed instead. However, the inclusion of effect sizes paired with the results of the *t*-tests enhanced confidence in the results as the effect sizes provided important conclusions on the magnitude and relevance of differences between means. Future research should consider recruiting larger, diversified numbers of coaches (e.g., >100) when using survey design studies of the TCW as more omnibus statistical analyses that control for potential covariates could be conducted (e.g., Multivariate Analysis of Variance). In line with the small sample size limiting the methodological aims of the research, future evaluations of the workshop may consider a more intensive program of study (e.g., longitudinal design). By making this adjustment, researchers can potentially gauge if the increased levels of engagement with the participants post-program contribute to significant findings in the future.

While the COM-B questionnaire was developed in collaboration with behavior change researchers and guided by the recommendations of Michie et al.,^32^ future research should aim further to test the reliability and validity of this instrument. For example, Confirmatory Factor Analyses should be conducted to confirm the construct validity of the sport-adapted COM-B questionnaire. Self-report measures can also pose limitations as they are subject to biases where participants can inflate their responses to be perceived in a socially desirable way.^50,51^ Future research may wish to include a scale in revisions of the COM-B questionnaire to control for social desirability effects (e.g., Marlowe-Crowne Social Desirability Scale^52^). Lastly, future evaluations of the workshop can look to utilize mixed-methods research designs whereby qualitative data (e.g., interview data) is collected alongside the quantitative data. Such qualitative data might also help future researchers determine if and how coach training programs can have a significant impact on the perceptions of the coaches who participate in interpersonal-focused CDPs.

### Theoretical and practical implications

Theoretically, this study used behavior change theory to examine an evidence-informed, interpersonal-focused CDP.^7^ Limited research exists that explicitly explores the application of behavior change frameworks to examine coaching programs despite their effective application for examining program effectiveness in non-sport domains (e.g., health promotion^25,26,27^). Using the COM-B measures, this research identifies specific antecedents of behavior that inform researchers on how the TCW impacted the coaches. Additionally, in their systematic review of PYD-focused sport programs, Williams et al.^53^ note that existing evaluative studies in the sport context overuse qualitative measures post-intervention and do not incorporate control groups to better contextualize changes observed post-program. The preference for qualitative approaches may be attributable to a lack of available, validated sport-specific measures for researchers, which is identified as a persisting issue in the sport literature.^53,54^ As such, this research advances the sport coaching science threefold: (a) provides a preliminary understanding of elements that influence the coaches’ use of transformational coaching behaviors,^22^ (b) uses a comparison group at pre-and post-intervention time points, and (c) demonstrates the novel application of behavior change theory to address a gap in the sport literature by proposing an alternative approach to evaluation in the sports science context.

Practically, this study contributes to the importance of collaboration with community sport organizations to examine the effectiveness of evidence-informed, interpersonal-focused coaching intervention. Using a quasi-experimental research design provides some insight into how coaches actual perceptions and behaviors are impacted by participation in a coaching intervention. Quasi-experimental designs in coaching research are important as they represent a marked difference from existing efficacy studies that are conducted in controlled conditions and are removed from the reality of coaching practice.^55^ For example, while observable behavior change is a primary goal of interventions, it is important that interventions also change coaches’ perceptions of behavior as this connects to long-term, sustained behavior change.^24^ In addition, the medium to large effect sizes for the intervention group results speak to the practical effect of the TCW on coaches’ perceptions post-workshop, suggesting that such findings can be useful and beneficial if this workshop were to be delivered to other coaches.^56^

Finally, behavior change theory can help sports science researchers better identify and understand factors that influence coaches’ behaviors (e.g., perceptions). The findings of this study build upon previous research evaluating the TCW^22^ and partially support the idea that interpersonal-focused CDPs, such as the TCW, can change the way coaches immediately perceive their behaviors. Such improved understanding may help inform what content and strategies researchers and practitioners should include in programs to better facilitate behavior change in coaches.^57^ Therefore, this study answers the Whitley et al.^58^ call for researchers to begin to test intervention theories as opposed to intervention outcomes to identify the conditions and mechanisms that explain observable changes in coaches’ behaviors.

## Conclusion

Vella et al.^18^ note that “it is still relatively unknown how actual coaching behavior is related to positive youth development when compared to perceived coaching behavior” (p. 527). Actual coaching behaviors are often mediated by athletes’ perceptions of the barriers and facilitators that influence their actions. Thus, this study addressed an important gap in the literature by examining the perceptual elements of behavior change (i.e., perceived capability, opportunity, and motivation) and how they are affected by participation in the TCW.^22^ As noted by Horn,^59^ the development of interventions to help change or modify coaches’ behaviors will be made easier once it is known *why* coaches exhibit the behaviors they do in the first place. Furthermore, given the shortage of validated measures of coaches’ perceptions of their behaviors,^59^ this study also provides support for future research to develop more rigorous instruments that use behavior change theory to capture how coaches perceive their behaviors. Further investigation into the use of COM-B measures in program examination with a larger sample may be warranted to continue exploring the effects of the interpersonal-focused CDPs on coaches’ behaviors.
